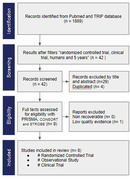# Comparison of cognitive behavioral therapy to delay the onset of dementia in patients with Alzheimer’s disease. A systematic Review

**DOI:** 10.1002/alz.087172

**Published:** 2025-01-09

**Authors:** Christan J Monge Ortega, Marcos Arreola Flores, Arid Barragán Ortíz, Sergio M Carrillo Rodríguez, Gilberto Mauricio Suárez, David A Chávez Castillo, Maria F Estrada Posadas, Sofia Andrade Lara, Thania F Estrada Ortega

**Affiliations:** ^1^ Universidad Autónoma de San Luis Potosi, San Luis Potosi, SL Mexico; ^2^ Universidad Autónoma del Estado de México, Toluca, EM Mexico

## Abstract

**Background:**

Following a diagnosis of dementia, the patient experiences changes that mainly affect his cognitive behavior. Various behavioral therapies are currently being explored to reduce these symptoms and improve the patient’s quality of life.

**Method:**

Flow chart

**Result:**

In the context of the emotional impact associated with the diagnosis of dementia, the search for interventions to mitigate emotional and social deterioration in patients has intensified.

Cognitive therapy (CT) and rTMSA altogether: 2 types of non‐pharmacological interventions rTMS and CT, in a research study one group together and in the other CT alone. Both groups improved their MMSE test scores, although people who received rTMS in conjunction with CT showed better test scores.

Cognitive Rehabilitation (CR): a clinical trial was conducted to evaluate the effectiveness of cognitive rehabilitation (CR), which consists of strategies to improve procedural learning of skills and to learn or relearn relevant information. Resulted in effective intervention for people with early Alzheimer’s disease.

Electronic Memory and Management Aid (EMMA): these therapies focus on a current intervention for older adults with Subjective Cognitive Risk (SCR), who maintain normal cognition, making the most of skill building and healthy habit formation. The research showed improvements in patients with early‐stage AD at 9 months. However, in more advanced stages, it proved ineffective.

Computerised cognitive training: it has started to show interesting evidence, although at present the evidence in favour remains weak and more robust studies are needed. Interestingly, the improvement was maintained at 6‐month follow‐up, but tended to decline at 12‐month follow‐up as baseline performance was reached.

**Conclusion:**

Concluding, extensive research is being carried out in the development of therapies aimed at mitigating cognitive disorders associated with Alzheimer’s disease. Among these, several are presented in this abstract, with applications that not only improve the quality of life but also the cognitive functioning of patients, proving to be effective, especially in the early stages of the disease.